# Epithelial to mesenchymal transition markers expressed in circulating tumour cells of early and metastatic breast cancer patients

**DOI:** 10.1186/bcr2896

**Published:** 2011-06-10

**Authors:** Galatea Kallergi, Maria A Papadaki, Eleni Politaki, Dimitris Mavroudis, Vassilis Georgoulias, Sophia Agelaki

**Affiliations:** 1 Laboratory of Τumor Cell Βiology, University of Crete, Voutes 71110, Heraklion, Crete, Greece; 2 Department of Medical Oncology, University of Crete, Voutes 71110, Heraklion, Crete, Greece

## Abstract

**Introduction:**

Epithelial to mesenchymal transition (EMT) is considered an essential process in the metastatic cascade. EMT is characterised by upregulation of vimentin, Twist, Snail, Slug and Sip1 among others. Metastasis is also associated with the presence of circulating tumour cells (CTCs) and disseminated tumour cells in the blood and bone marrow, respectively, of breast cancer patients, but the expression of EMT markers in these cells has not been reported so far.

**Methods:**

The expression of Twist and vimentin in CTCs of 25 metastatic and 25 early breast cancer patients was investigated by using double-immunofluorescence experiments in isolated peripheral blood mononuclear cell cytospins using anti-cytokeratin (anti-CK) anti-mouse (A45-B/B3) and anti-Twist or anti-vimentin anti-rabbit antibodies.

**Results:**

Among early breast cancer patients, vimentin-and Twist-expressing CK^+ ^CTCs were identified in 77% and 73% of the patients, respectively, and in 100% of the patients with metastatic breast cancer for both markers (*P *= 0.004 and *P *= 0.037, respectively). Among patients with early disease, 56% and 53% of the CK^+ ^CTCs were double-stained with vimentin and Twist, and the corresponding values for metastatic patients were 74% and 97%, respectively (*P *= 0.005 and *P *= 0.0001, respectively). The median expression of CK^+^vimentin^+ ^and CK^+^Twist^+ ^cells per patient in metastatic patients was 98% and 100%, and in an adjuvant chemotherapy setting the corresponding numbers were 56% and 40.6%, respectively. Triple-staining experiments revealed that all CK^+^Twist^+ ^or CK^+^vimentin^+ ^cells were also CD45^-^, confirming their epithelial origin. Immunomagnetic separation of CTCs and triple-immunofluorescence with anti-CK/anti-Twist/anti-vimentin antibodies demonstrated that both mesenchymal markers could be coexpressed in the same CK^+ ^cell, since 64% of the total identified CTCs were triple-stained. There was a significant correlation (*P *= 0.005) between the number of CTCs expressing Twist and vimentin within the same setting.

**Conclusions:**

CTCs expressing Twist and vimentin, suggestive of EMT, are identified in patients with breast cancer. The high incidence of these cells in patients with metastatic disease compared to early stage breast cancer strongly supports the notion that EMT is involved in the metastatic potential of CTCs.

## Introduction

Metastasis is associated with the presence of peripheral blood circulating tumour cells (CTCs) and bone marrow disseminated tumour cells (DTCs) in patients with breast cancer [1,2]. In fact the presence of CTCs before the initiation and after the completion of adjuvant chemotherapy is associated with poor clinical outcome [3-5]. In metastatic breast cancer, the assessment of CTCs before and shortly after the initiation of chemotherapy is also predictive of progression-free and overall survival [6,7], and prognosis seems to depend on the detection of CTCs rather than DTCs [8]. The presence of chromosomal alterations confirmed the malignant nature of CTCs [9,10]. Nevertheless, only some of them are capable of promoting metastasis [11]. Therefore, further molecular characterisation of CTCs is essential to understanding their metastatic potential, as well as for the identification of additional markers related to patients' prognosis.

The metastatic process consists of distinct steps, including tumour growth, angiogenesis, tumour cell detachment, epithelial to mesenchymal transition (EMT), intravasation, survival within blood and lymphatic vessels and embolisation, extravasation, mesenchymal to epithelial transition, formation of micrometastasis and, finally, growth of macrometastasis [12]. ΕΜΤ is a process whereby epithelial cells lose their epithelial characteristics and acquire a mesenchymal phenotype. EMT increases the metastatic and invasive potential of these cells [13]. Downregulation of epithelial markers such as cytokeratin and E-cadherin and upregulation of mesenchymal markers such as vimentin, N-cadherin and cadherin 11 characterise the EMT process. Usually, inhibition of E-cadherin expression leads to induction of N-cadherin expression, which has been associated with tumour invasiveness [14-16]. Transforming growth factor β as well as transcription factors such as Twist, Snail, Slug and Sip1 have a regulatory role in EMT.

Twist is a transcriptional repressor of the E-cadherin gene [12,17]. Increased expression of Twist has been demonstrated in many types of tumour cells, such as melanoma, osteosarcoma, T cells (Sézary syndrome) and gastric, prostate and breast cancer [12,18-23]. The gene expression profile of immunomagnetically isolated DTCs has shown elevated expression of Twist in the enriched fragment compared to that in healthy volunteers [24,25]. Twist expression in breast cancer cells has been shown to result in resistance to paclitaxel through binding to Akt promoter and enhancement of its transcriptional activity [26], as well as resistance to other microtubule-targeting agents such as vincristine [27,28]. Upregulation of Twist in cancer cells increases vascular endothelial growth factor (VEGF) gene expression [21,29], while hypoxia-inducible factor 1α (HIF-1α) regulates the expression of Twist by binding directly to the hypoxia response element in the Twist proximal promoter [30,31]. We have recently shown that VEGF and HIF-1α, as well as phosphorylated Akt, are expressed in CTCs of most metastatic breast cancer patients [32,33]; therefore, it was interesting to further investigate the expression of Twist in CTCs of breast cancer patients.

Vimentin is an intermediate filament normally expressed in mesenchymal cells and is involved in the migration of epithelial cells during development [34]. The expression of vimentin in cancer cells is believed to enhance migration and invasiveness [35]. Expression of vimentin is characteristic of epithelial cells undergoing the EMT process and is related to reduced expression of E-cadherin and upregulation of N-cadherin [16,36], while increased expression of vimentin in breast carcinomas is correlated with poor prognosis [37]. Moreover, the simultaneous expression of vimentin and cytokeratin in tumour cells is associated with poorer survival in breast cancer patients [38]. Recent studies have demonstrated that vimentin is expressed in DTCs of breast cancer patients and tumour cell lines [39,40]; however, there have been no studies in which the expression of both EMT markers (Twist and vimentin) was evaluated in CTCs. Therefore, the aim of the present study was to investigate the expression of these molecules in CTCs of patients with early and metastatic breast cancer.

## Materials and methods

### Patient samples and cytospin preparation

A longitudinal trial for the study of micrometastatic disease in breast cancer has been underway in our institution since 1996. Peripheral blood samples are obtained from patients who provide their written informed consent as part of the routine evaluation before the initiation of and at the end of adjuvant treatment, as well as prior to and after the completion of each chemotherapy line in patients with metastatic disease. Frozen RNA samples and peripheral blood mononuclear cell (PBMC) cytospins are prepared simultaneously and stored at -80°C until use. In the current trial, samples taken from patients with adjuvant or metastatic breast cancer were screened for cytokeratin (CK)-19 mRNA expression [41,42] by RT-PCR. Fifty CK-19 mRNA-positive patients (25 with early stage breast cancer and 25 with metastatic breast cancer) were enrolled in the present study. We used archived slides from these patients because we wanted to have the same blood samples for real-time RT-PCR and immunocytochemistry. Ten healthy female blood donors were also included as a control group. All blood samples were obtained at the middle of venipuncture after the first 5 mL of blood were discarded. These precautions were undertaken to avoid contamination of the blood samples with epithelial cells from the skin during sample collection. All patients and healthy volunteers gave their written informed consent to participate in the study, which has been approved by the Ethics and Scientific Committees of our institution.

The volume of blood drawn from each patient was 20 mL each for immunofluorescence and RT-PCR experiments and 20 mL for immunomagnetic isolation. PBMCs were isolated by Ficoll-Hypaque density gradient centrifugation (*d *= 1,077 g/mol) at 1,800 rpm for 30 minutes. PBMCs were washed three times with PBS and centrifuged at 1500 rpm for 10 minutes. Aliquots of 250,000 cells were cytocentrifuged at 2,000 rpm for 2 minutes on glass slides. Cytospins were dried up and stored at -80°C. Four to five slides from each patient were used for staining experiments, so 1 × 10^6 ^PBMCs per patient were scanned.

### Cell cultures

In control experiments, we used the human cervical adenocarcinoma cell line HeLa. HeLa cells express Twist and vimentin and have been proposed as a positive control by the antibody's data sheet. The HeLa adenocarcinoma cells (American Type Culture Collection, Manassas, VA, USA) were cultured in 1:1 (vol/vol) DMEM (Gibco-BRL, Grand Island, NY) supplemented with 10% foetal bovine serum (FBS) (Gibco-BRL), 2 mmol L-glutamine (Gibco-BRL) and 50 mg/mL penicillin/streptomycin (Gibco-BRL). Cells were maintained in a humidified atmosphere of 5% CO_2 _and 95% room air. Subcultivation of all cell lines was performed using 0.25% trypsin and 5 mmol ethylenediaminetetraacetic acid (EDTA) (Gibco-BRL). All experiments were performed during the logarithmic growth phase 15 to 20 hours prior to the experiments. HeLa cells were spiked in blood obtained from healthy volunteers, and cytospins were prepared afterward with Ficoll-Hypaque density gradient centrifugation as per patients' samples.

### Double-immunofluorescence confocal laser-scanning and ARIOL scanning microscopy

The presence of CK-positive cells in PBMC cytospin preparations was investigated using the mouse A45-B/B3 antibody (detecting CK8, CK18 and CK19) (Micromet, Munich, Germany). Control experiments for the sensitivity and the specificity of this antibody have been reported previously [32,33,43]. The cytomorphological criteria proposed by Meng *et al. *[44] (for example, high nuclear/cytoplasmic ratio, larger cells than white blood cells) were used to characterise a CK-positive cell as a CTC.

Cytospins from the same patients were double-stained for Twist/CK (Abcam, Cambridge, UK) and vimentin/CK (Santa Cruz Biotechnology, Santa Cruz, CA, USA) in double-staining experiments [43]. Specific staining can easily be distinguished by double-immunofluorescence because of the differential intracellular distribution of the examined molecules compared to nonspecific staining as reported by Fehm *et al. *[45]. For double-staining experiments, PBMC cytospins were fixed with 3% paraformaldehyde. Permeabilisation of the cell membrane was performed with 0.5% Triton for 10 min and blocking with PBS/1% BSA overnight. Subsequently, slides were stained for cytokeratin with the A45-B/B3 anti-mouse antibody along with the corresponding secondary fluorescein isothiocyanate (FITC) fluorochrome. Slides were then stained with Twist or vimentin anti-rabbit antibodies and afterward with the corresponding anti-rabbit secondary antibodies for 45 minutes. Negative controls were performed for all the primary antibodies by omitting the corresponding primary antibody and adding the secondary immunoglobulin G (IgG) isotype antibody. Finally, 4',6-diamidino-2-phenylindole (DAPI) antifade reagent (Invitrogen, Carlsbad, CA, USA) was added to each sample for nuclear staining. Slides were analysed using a confocal laser-scanning microscope (Leica Lasertechnik, Heidelberg, Germany) and the ARIOL CTC automated image analysis system (Genetix, New Milton, Hampshire, UK).

### Triple immunofluorescence

Triple-immunofluorescence for CK/Twist/CD45, CK/vimentin/CD45 and CK/Twist/vimentin was also performed in samples processed by immunomagnetic separation to be enriched in CTCs. Cells were initially fixed using 4% formaldehyde for 15 minutes at room temperature. Permeabilisation was achieved with 0.1% Triton X-100 for 5 minutes at room temperature. After blocking with PBS supplemented with 10% (vol/vol) FBS for 30 minutes, cells were incubated with the corresponding antibodies for 45 minutes each. Zenon technology (FITC-conjugated IgG1 antibody) (Molecular Probes/Invitrogen, Carlsbad, CA, USA) was used for CK detection with the A45-B/B3 antibody. Zenon antibodies were prepared within 30 minutes of being used.

**Figure 1:**
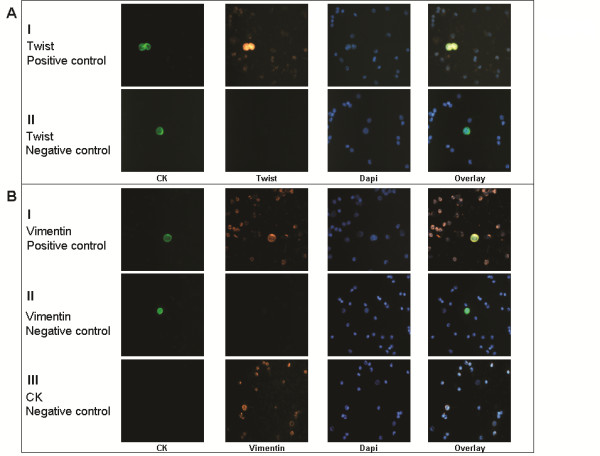
**Twist and vimentin expression in HeLa cells spiked in blood of normal volunteers**. ARIOL system images of HeLa cells spiked in the blood of normal volunteers. **(A) **Row I: Positive control for Twist. HeLa cells were stained with pan-CK A45-B/B3 antibody/secondary FITC anti-mouse antibody (green)/Twist anti-rabbit antibody/Alexa Fluor 555 anti-rabbit antibody (orange). Row II: Negative control for Twist. Cells were stained with pan-CK A45-B/B3 antibody/FITC anti-mouse (green) and Alexa Fluor 555 IgG isotype antibody. Cell nuclei were stained with DAPI (blue). Original magnification, x400. **(B) **Row I: Positive control for vimentin. Cells were stained with pan-CK A45-B/B3 antibody/FITC anti-mouse antibody (green)/vimentin anti-rabbit antibody/Alexa Fluor 555 anti-rabbit antibody (orange). Row II: Negative control for vimentin. Cells were stained with pan-CK A45-B/B3 antibody/FITC anti-mouse antibody (green) and Alexa Fluor 555 IgG isotype antibody. Row III: Negative control for pan-CK. Cells were stained with FITC IgG isotype antibody/vimentin anti-rabbit antibody/Alexa Fluor 555 anti-rabbit antibody (orange). Cell nuclei were stained with DAPI (blue). Original magnification, x400. ARIOL system = automated image analysis system; CK = cytokeratin; FITC = fluorescein isothiocyanate; HeLa = patient with cervical adenocarcinoma from which the cell line was derived; IgG = immunoglobulin G; DAPI = 4',6-diamidino-2-phenylindole.

Twist was detected using anti-mouse antibody (Abcam) labelled with Alexa Fluor 633 (Molecular Probes/Invitrogen) or Twist anti-rabbit (Cell Signaling Technology, Boston, MA, USA) labelled with Alexa Fluor 555 (Molecular Probes, Carlsbad, CA, USA). Positive and negative controls for Twist anti-rabbit are shown in Figure 1A, while positive and negative controls for the Twist anti-mouse antibody are presented in Additional file 1. Vimentin was detected using anti-rabbit antibody (Santa Cruz Biotechnology) labelled with Alexa Fluor 555, and positive and negative controls are shown in Additional file 1. CD45 was detected with an anti-mouse antibody (Dako, Carpinteria, CA, USA) labelled with Alexa Fluor 633. Cells incubated with the different antibodies were postfixed with 4% (vol/vol) formaldehyde in PBS for 15 minutes at room temperature. Finally, cells were stained with DAPI conjugated with antifade reagent.

### Immunomagnetic separation of CTCs

Α negative selection procedure was used for the isolation of CTCs according to the method described by Naume *et al. *[46]. A quantity of 100 μL of Dynal CELLection beads (Invitrogen) coated with anti-CD45 monoclonal antibody were added to 1 × 10^7P^/mL PBMCs in PBS/0.1% BSA/2 mM EDTA. After incubation for 30 minutes at 4°C, the supernatant was transferred into FBS-coated tubes and cells were cytocentrifuged at 2,000 rpm for 2 minutes on glass slides. The same number of cells was centrifuged at 1,500 rpm for 5 minutes, and the pellet was stored at -80°C for RNA extraction. The specificity and the sensitivity of this method has been described previously [33]. CD45 immunomagnetic depletion was performed for the purpose of triple-staining experiments only to enrich the samples with CTCs.

## Results

### Twist and vimentin expression in HeLa cells and PBMCs of normal blood donors

HeLa cell cytospins spiked in blood from healthy volunteers and processed as per patient samples were used as positive controls for the detection of Twist and vimentin. Positive and negative controls for Twist, vimentin and CK are shown in Figure 1.

Twist and vimentin expression was subsequently investigated in PBMC cytospins from 10 healthy blood donors. Both molecules were found to be expressed spontaneously in PBMCs, but there were no double-positive cells (CK^+^Twist^+ ^or CK^+^vimentin^+^) in healthy volunteers.

### Twist and vimentin expression on CTCs of early stage and metastatic breast cancer patients

Twenty-five patients with metastatic breast cancer and twenty-five patients with early breast cancer who had detectable CK-19 mRNA-positive cells by RT-PCR were enrolled in this study. The concordance between RT-PCR and immunocytochemistry was 100% in metastatic breast cancer patients and 88% in early breast cancer patients. The clinicopathological characteristics of these patients are presented in Table 1.

**Table 1:** Patient characteristics^a^

Early breast cancer	Metastatic breast cancer
Number of patients enrolled = 25	Number of patients enrolled = 25
Age, years	Age, years
Median (range), 59 (26 to 76)	Median (range), 59 (36 to 83)
ECOG performance status, *n *(%)	ECOG performance status, *n *(%)
0 24 (96%)	0 8 (32%)
1 1 (4%)	1 13 (52%)
2 0 (0%)	2 4 (16%)
Histology, *n *(%)	Histology, *n *(%)
Ductal 22 (88%)	Ductal 20 (80%)
Lobular 3 (12%)	Lobular 1 (4%)
Other 0 (0%)	Unknown 4 (16%)
Menopausal status, *n *(%)	Menopausal status, *n *(%)
Premenopausal 8 (32%)	Premenopausal 4 (16%)
Perimenopausal 1 (4%)	Perimenopausal 1 (4)
Postmenopausal 16 (64%)	Postmenopausal 20 (80%)
Hormone receptor status, *n *(%)	Hormone receptor status, *n *(%)
ER-positive/PR-positive 11 (44%)	ER-positive/PR-positive 11 (44%)
ER-positive/PR-negative 6 (24%)	ER-positive/PR-negative 3 (12%)
ER-negative/PR-positive 1 (4%)	ER-negative/PR-positive 2 (8%)
ER-negative/PR-negative 7 (28%)	ER-negative/PR-negative 9 (36%)
Tumour size, *n *(%)	Number of disease sites, *n *(%)
1 to 1.9 cm (T1) 6 (24%)	1 7 (28%)
2 to 5 cm (T2) 16 (64%)	2 9 (36%)
>5 cm (T3) 1 (4%)	3 4 (16%)
Unknown 2 (8%)	≥4 5 (20%)
Tumour grade, *n *(%)	Line of treatment, *n *(%)
I 2 (8%)	First 11 (44%)
II 9 (36%)	Second 7 (28%)
III 13 (52%)	≥Third 7 (28%)
Unknown 1 (4%)	
	Primary breast cancer at presentation, *n *(%)
	Early 9 (36%)
Positive nodes, *n *(%)	Metastatic 16 (64%)
0 (N0) 11 (44%)	
1 to 3 (N1) 9 (36%)	Visceral disease, *n *(%)
4 to 9 (N2) 2 (8%)	Yes 13 (52%)
≥10 (N3) 2 (8%)	No 12 (48%)

^a^ECOG = Eastern Cooperative Oncology Group; ER = oestrogen receptor; PR = progesterone receptor.

**Figure 2:**
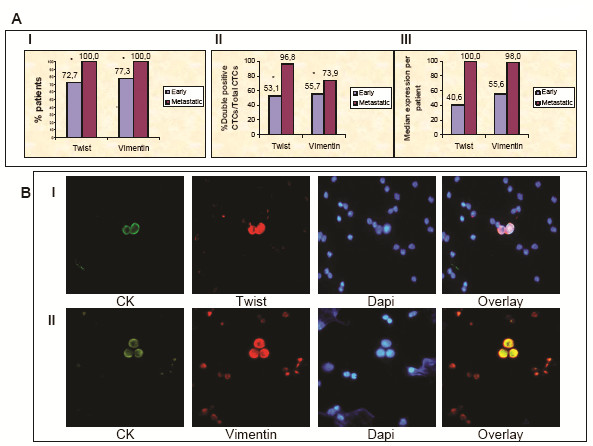
**Twist and vimentin expression in CTCs of early and metastatic breast cancer patients**. **(A) **Graph I: Quantification of 25 early and 25 metastatic breast cancer patients in whom double-positive cells of each examined molecule were harvested. Graph II: Quantification of double-positive CTCs/total CTCs for each examined molecule. Graph III: Quantification of median expression per patient for each examined molecule. **(B) **Representative ARIOL system images of CTCs in PBMC cytospin. Row I: Cytospin double-stained with monoclonal pan-CK A45-B/B3 (green)/polyclonal Twist anti-rabbit (red) antibodies and DAPI nuclear staining. Original magnification, x400. Row II: Cytospin double-stained with monoclonal A45-B/B3 (green)/polyclonal vimentin anti-rabbit (red) antibodies and DAPI nuclear staining. Original magnification, x400. ARIOL system = automated image analysis system; CK = cytokeratin; CTCs = circulating tumour cells; PBMCs = peripheral blood mononuclear cells; DAPI = 4',6-diamidino-2-phenylindole.

**Figure 3:**
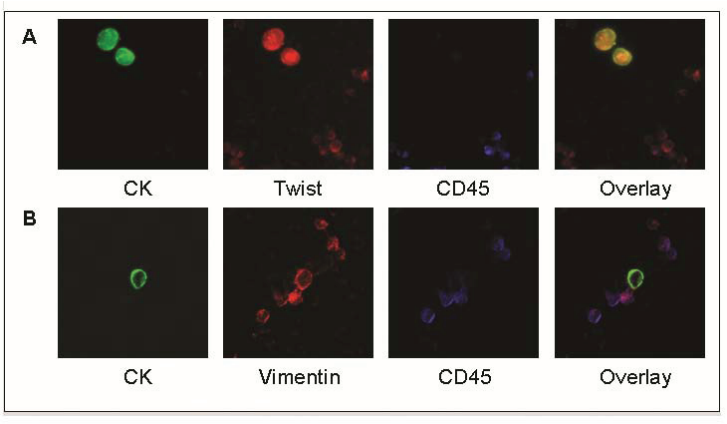
**Triple-immunofluorescence (CK/Twist/CD45, CK/vimentin/CD45) in CTCs**. Representative ARIOL system photomicrographs of CTC cytospin after negative immunomagnetic separation in patients with metastatic breast cancer. **(A) **Cells were triple-stained with pan-CK A45-B/B3 antibody/Zenon Alexa Fluor 488 (green)/Twist anti-rabbit antibody/Alexa Fluor 555 anti-rabbit antibody (orange) and CD45 anti-mouse/Alexa Fluor 633 anti-mouse antibody (blue). Original magnification, x400. **(B) **Cells were triple-stained with pan-CK A45-B/B3 antibody/Zenon-Alexa Fluor 488 (green)/vimentin anti-rabbit antibody/Alexa Fluor 555 anti-rabbit antibody (orange) and CD45 anti-mouse/Alexa Fluor 633 anti-mouse antibody (blue). Original magnification, x400. ARIOL system = automated image analysis system; CK = cytokeratin; CTCs = circulating tumour cells.

**Figure 4:**
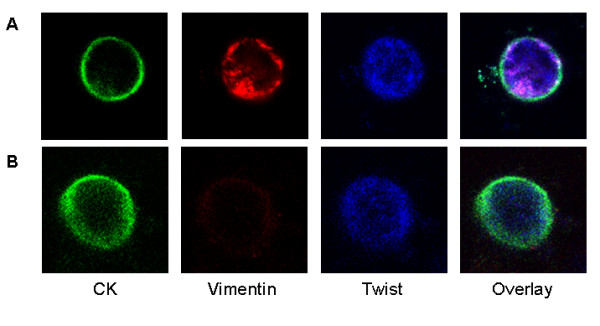
**Coexpression of CK, Twist and vimentin in the same cell**. Representative confocal laser-scanning photomicrographs of CTC cytospin after negative immunomagnetic separation in a patient with metastatic breast cancer. Cells were triple-stained with pan-CK A45-B/B3 antibody/Zenon Alexa Fluor 488 (green)/Twist anti-mouse/Alexa Fluor 633 anti-mouse antibody/vimentin anti-rabbit/Alexa Fluor 555 anti-rabbit antibody (orange). Original magnification, x600. **(A) **A CTC expressing CK, Twist and vimentin. **(B) **A CTC expressing CK and Twist, but not vimentin. CK = cytokeratin; CTCs = circulating tumour cells.

We investigated the expression of Twist and vimentin on CTCs using PBMC cytospin preparations from these patients. The presence of CK^+ ^cells was confirmed by immunofluorescence in all patients with metastatic disease and in 22 of 25 patients with early stage disease. Double-staining experiments with pan-CK and Twist antibodies revealed that all patients with metastatic disease had detectable double-stained cells and that CK^+^/Twist^+ ^cells could be detected in 16 (72.7%) of 22 patients with early stage breast cancer (*P *= 0.037) (Figure 2A, graph I, and Table 2). Additionally, the proportion of double-positive CTCs was lower in patients with early stage breast cancer than in patients with metastatic breast cancer (53% versus 97%, respectively; *P *= 0.0001) (Figure 2A, graph II). Confocal laser-scanning microscopy and ARIOL system microscopy revealed that Twist was located both in the cytoplasm and in the nuclei of CTCs (Figures 2B, graph I; Figures 3 and 4; and Additional file 2). The median number of CTCs per patient expressing Twist^+^/CK^+ ^was 100% (range, 33% to 100%) in patients with metastatic disease and 40.6% (range, 12% to 100%) in patients with early stage breast cancer (Figure 2A, graph III). Nineteen patients with metastatic breast cancer (76%) had only double-positive cells (Twist^+^CK^+^), while in early breast cancer patients only six (27%) of twenty-two CK^+ ^patients had exclusively Twist^+^CK^+ ^cells (*P *= 0.0001) (Table 2).

**Table 2:** Number of double-stained CTCs/250,000 PBMCs in early and metastatic breast cancer patients^a^

Early breast cancer					Metastatic breast cancer				
**Patient**	**Vim^+^CK^+^**	**Vim^-^CK^+^**	**Twist^+^CK^+^**	**Twist^-^CK^+^**	**Patient**	**Vim^+^CK^+^**	**Vim^-^CK^+^**	**Twist^+^CK^+^**	**Twist^-^CK^+^**

1	64	51	83	100	1	5	0	4	0
2	25	63	29	142	2	45	0	8	0
3	31	111	578	68	3	21	0	7	0
4	35	28	53	85	4	2	0	4	0
5	135	199	18	134	5	74	20	45	0
6	100	158	28	170	6	7	7	3	6
7	170	254	89	423	7	7	2	420	0
8	0	0	74	4	8	21	69	2	0
9	0	1	71	95	9	10	90	3	0
10	140	4	0	1	10	2	0	2	0
11	22	0	0	0	11	3	15	103	36
12	17	0	74	88	12	67	3	9	1
13	0	4	8	0	13	4	0	0	0
14	0	0	0	3	14	3	3	42	0
15	7	1	0	4	15	1	0	1	0
16	10	3	12	0	16	1	0	1	0
17	0	0	0	0	17	10	2	55	0
18	28	12	0	0	18	325	97	5	7
19	0	0	0	10	19	17	1	13	0
20	0	1	2	0	20	3	0	0	0
21	2	0	13	0	21	2	0	2	0
22	2	0	0	9	22	2	1	2	0
23	306	0	380	0	23	9	0	658	0
24	1	0	0	2	24	192	4	44	0
25	24	0	1	0	25	40	0	80	0

^a^CTCs = circulating tumour cells; PBMCs = peripheral blood mononuclear cells; Vim = vimentin; CK = cytokeratin.

We subsequently assessed the expression of vimentin in CTCs of the same cohort of patients. Vimentin was also expressed in CTCs in 100% and 77% of patients with metastatic and early stage disease, respectively (*P *= 0.004) (Figure 2A, graph I, and Table 2). Furthermore, the proportion of double-positive CTCs was lower in patients with early stage cancer than in those with metastatic disease (56% versus 74%; *P *= 0.005) (Figure 2A, graph II). In addition, the median proportion of double-positive CTCs per patient with early stage and metastatic disease was 56% (range, 21.8% to 100%) and 98% (range, 16.7% to 100%), respectively (Figure 2A, graph III). In 12 (48%) of 25 patients with metastatic disease, all the CTCs were double-positive (vimentin^+ ^CK^+^) compared with only 6 (27%) of 22 patients with early disease who had exclusively vimentin^+^CK^+ ^CTCs (*P *= 0.048) (Table 2). Confocal laser-scanning microscopy and ARIOL system microscopy revealed that the intracellular distribution of vimentin in CTCs was almost identical to that of cytokeratin filaments (Figure 2B, graph II; Figures 3 and 4; and Additional file 2).

A statistically significant correlation (Spearman's ρ analysis) was also observed between the number of CTCs expressing Twist and vimentin in early stage (*P *= 0.027) and metastatic (*P *= 0.009) breast cancer patients. This correlation remained significant even when all patients were grouped together (*P *= 0.005).

### Triple-immunofluorescence experiments revealed specific expression of Twist and vimentin in CTCs

As mentioned above, Twist and vimentin expression was also observed in PBMCs. To confirm that the cells characterised as CTCs in breast cancer patients were nonhematopoietic cells presenting ectopic cytokeratin expression, we performed triple-immunofluorescence experiments using antibodies against CKs and CD45 along with antibodies against Twist or vimentin. These experiments were carried out indicatively in five metastatic and eight early breast cancer patients. Our results revealed that no CK^+^vimentin^+^CD45^+ ^or CK^+^Twist^+^CD45^+ ^cells could be identified in patient samples (Figure 3).

### Vimentin and Twist are coexpressed in CTCs of breast cancer patients

Subsequently, we investigated the possible coexpression of the examined mesenchymal markers in the same CTC. After performing CD45^- ^immunomagnetic separation in PBMCs isolated from 24 patients with metastatic disease, the CTC-enriched fraction was cytocentrifuged on one slide for each patient. Triple-immunofluorescence experiments using anti-pan-CK, anti-Twist and anti-vimentin antibodies revealed that 16 (64%) of 25 CTCs identified were CK^+^Twist^+^vimentin^+ ^and 9 (36%) of 25 CTCs identified were CK^+^Twist^+^vimentin^-^. None of the detected CTCs had the CK^+^Twist^-^vimentin^+ ^phenotype. These results indicate that Twist and vimentin are coexpressed in a subset of CTCs of metastatic breast cancer patients (Figure 4).

## Discussion

There is increasing evidence that the presence of CTCs and DTCs is correlated with minimal residual disease or disease progression in patients with breast cancer. Nevertheless, the underlying molecular characteristics of micrometastatic cells associated with the development of overt metastases remain largely unknown. EMT is a multistep process that has been suggested to play a key role in cancer progression and metastasis [12]. Accordingly, CTCs bearing characteristics of an EMT phenotype should be actively involved in tumour dissemination, proliferation and metastasis. Twist is a transcription factor that, among others, participates in EMT and is upregulated in many tumour cells [18-22,47]. In a recent report by Watson *et al. *[24], Twist expression was specifically enhanced in a gene signature obtained from epithelial cell adhesion molecule-enriched bone marrow samples of patients with breast cancer after neoadjuvant chemotherapy. Twist also increases VEGF expression, while it is directly regulated by HIF-1α [30,31]. Since we have recently shown that 62% and 76% of breast cancer patients express VEGF and HIF-1α, respectively, in their CTCs [33], it was of interest to identify the potential expression of this transcription factor in CTCs.

In the present cytomorphological study, we compared for the first time the expression of Twist and vimentin in individual CTCs of patients with early stage versus metastatic breast cancer. For this purpose, we performed double-staining experiments with the corresponding antibodies in PBMC cytospin preparations of 25 early stage and 25 metastatic breast cancer patients. To confirm the specificity of our results, we also performed triple-staining experiments with pan-CK/Twist/CD45 and pan-CK/vimentin/CD45 antibodies, which revealed that the hematopoietic antigen CD45 was not expressed in CK^+^/Twist^+ ^or CK^+^/vimentin^+ ^cells. These findings clearly indicate that the observed expression of Twist and vimentin is not confined to hematopoietic cells.

Double-staining experiments revealed that Twist was expressed in all CK-19 mRNA^+ ^metastatic patients, while most of them (76%) had exclusively double-positive cells (CK^+^Twist^+^). The median expression per patient of Twist in CTCs was also high at 100%. Similar results have also been reported in two recent studies by Aktas *et al. *[48] and Mego *et al. *[49]. In these studies, EMT markers including Twist were detected in CTCs of breast cancer patients at lower percentages of 42%.and 57.7%, respectively. This lower level of expression might be related to the difference in the identification method or to immunomagnetic enrichment of tumour cells.

Interestingly, when vimentin expression was investigated in the same cohort of patients, it was observed that, similarly to Twist, vimentin was expressed in CTCs of all the evaluated patients with metastatic disease, and the proportion of patients who had exclusively double-positive cells (CK^+^vimentin^+^) was also high. The median percentage of vimentin-expressing CTCs per patient was 98%. Vimentin is an intermediate filament of mesenchymal cells that is commonly used to identify cells undergoing EMT in cancer. In addition, its expression is associated with increased risk of metastasis and poor prognosis in breast cancer patients [37,38]. There are only two previous reports verifying the expression of vimentin in DTC cell lines and in CTCs [38,50]. In agreement with these data, the high rate of Twist and vimentin expression in CTCs observed in our study implies that mesenchymal markers are highly expressed in CTCs of metastatic breast cancer patients and denotes that the majority of these cells are undergoing the EMT process.

Furthermore, our experiments revealed that CK^+^Twist^+ ^and CK^+^vimentin^+ ^CTCs were also observed in patients with early stage breast cancer. This observation suggests that circulating epithelial (CK^+^) cells with an EMT phenotype may be involved in the continuous tumour spreading in patients with clinically undetectable metastases. However, the EMT phenotype expression was significantly lower (*P *= 0.037 for Twist and *P *= 0.004 for vimentin) in early stage cancer patients than in metastatic breast cancer patients. The median expression of Twist and vimentin was also significantly lower (40.6% and 55.6%, respectively) in early stage cancer patients, but it was extremely variable from patient to patient (ranges, 11% to 100% and 21% to 100%, respectively). The proportion of double-positive CTCs in early stage cancer patients was 53% and 56% for Twist and vimentin, respectively, which was also significantly different from metastatic breast cancer patients (*P *= 0.0001 and *P *= 0.05, respectively). These findings clearly indicate that EMT markers may be expressed in CTCs of patients with early stage disease, but to a lesser extent than in patients with metastatic disease. In addition, the above results imply that the population of CTCs expressing EMT markers predominates during disease progression or, alternatively, this cell population is selected over non-EMT-expressing CTCs because of resistance to therapy. However, this finding should be interpreted with caution because of the smaller number of CTCs detected in patients with early stage compared to advanced disease, which might influence the chance to find an EMT-like CTC.

A statistically significant correlation between the number of CTCs expressing Twist and vimentin in patients with both early stage and metastatic disease was demonstrated (*P *= 0.005 and *P *= 0.027, respectively), suggesting that both biomarkers were simultaneously expressed in CTCs. This assumption was confirmed by triple-staining experiments using antibodies against CK, Twist and vimentin in immunomagnetically isolated CTCs from 24 patients, which demonstrated that the majority (64%) of CTCs had the CK^+^Twist^+^vimentin^+ ^phenotype. Nevertheless, 36% of the CTCs were CK^+^Twist^+^vimentin^-^, whereas there were no cells with the CK^+^Twist-vimentin^+ ^phenotype (Figure 4). These findings indicate the heterogeneity of CTCs. It is also possible that Twist expression is a more common phenomenon during EMT, thus suggesting Twist as a more specific marker for EMT.

Finally, a differential intracellular distribution for vimentin and Twist was observed in CTCs. Vimentin was primarily located in the cytoplasm, similarly to the intracellular distribution of CK (Figures 2, 3, 4). This observation is in agreement with the findings of previous reports showing that vimentin filaments follow the preexisting CK network during EMT progression [51]. Twist showed both cytoplasmic and nuclear localisation as expected, considering its function as a transcription factor (Figures 2, 3, 4).

## Conclusions

The results of the present study clearly indicate that EMT markers such as Twist and vimentin are expressed in CTCs of patients with early stage and metastatic breast cancer. The variable expression of these molecules in CTCs at different stages of disease implies the predominance of the EMT phenotype during disease evolution. This hypothesis is further supported by the observation that CK^+ ^CTCs in patients with early stage breast cancer are more heterogeneous with regard to the expression of EMT markers, necessitating additional studies to further elucidate their distinct biological role.

## Abbreviations

ARIOL system: automated image analysis system; BSA: bovine serum albumin; CTCs: circulating tumour cells; DMEM: Dulbecco's modified Eagle's medium; DTCs: disseminated tumour cells; ECOG: Eastern Cooperative Oncology Group; EMT: epithelial-to-mesenchymal transition; FITC: fluorescein isothiocyanate; HeLa: patient with cervical adenocarcinoma from which the cell line was derived; PBMCs: peripheral blood mononuclear cells; PBS: phosphate-buffered saline; RT-PCR: reverse transcriptase polymerase chain reaction.

## Competing interests

The authors declare that they have no competing interests.

## Authors' contributions

GK participated in the design and coordination of the study, performed the immunomagnetic separations and the cell cultures and drafted the manuscript. MP and EP performed the immunofluorescence experiments and were involved in drafting the manuscript. SA helped draft the manuscript and collected all the clinicopathological characteristics of the patients. DM helped to draft the manuscript and participated in study design. VG provided general support, participated in study design and was involved in drafting the manuscript. All the authors gave their final approval of the version to be published.

## Supplementary Material

Additional file 1 **Triple-staining control experiments in HeLa cells**. Triple-staining control experiments in HeLa cells analysed using a confocal laser-scanning microscope. (I) Representative confocal laser-scanning photomicrographs of positive controls for Twist anti-mouse antibody. HeLa cells were stained with pan-CK A45-B/B3 antibody/Zenon Alexa Fluor 488 (green)/Twist anti-mouse/Alexa Fluor 633 anti-mouse IgG (blue)/vimentin anti-rabbit antibody/Alexa Fluor 555 anti-rabbit antibody (red). Original magnification, x500. (II) Negative control for Twist. HeLa cells stained with pan-CK A45-B/B3 antibody/Zenon Alexa Fluor 488 (green)/Alexa Fluor 633 anti-mouse IgG (blue)/vimentin anti-rabbit antibody/Alexa Fluor 555 anti-rabbit antibody (red). Original magnification, x500. (II) Negative control for vimentin. HeLa cells were stained with pan-CK A45-B/B3 antibody/Zenon Alexa Fluor 488 (green)/Twist anti-mouse antibody/Alexa Fluor 633 anti-mouse antibody (blue)/Alexa Fluor 555 IgG isotype antibody (red). Original magnification, x500. CK = cytokeratin; HeLa = patient with cervical adenocarcinoma, from which the cell line was derived; IgG, immunoglobulin G. Click here for file

Additional file 2 **Twist and vimentin expression in CTCs of breast cancer patients**. Representative confocal laser-scanning microscopic images of Twist-or vimentin-expressing CTCs of breast cancer patients. (I) Representative confocal laser-scanning microscopic images of breast cancer patients' cytospin double-stained with monoclonal pan-CK (A45-B/B3)/Alexa Fluor 488 anti-mouse antibody (green)/polyclonal Twist anti-rabbit antibody and Alexa Fluor 555 anti-rabbit antibody (red). Original magnification, x600. (II) Representative images of cytospin double-stained with monoclonal pan-CK (A45-B/B3)/Alexa Fluor 488 anti-mouse antibody (green)/polyclonal vimentin anti-rabbit antibody and Alexa Fluor 555 anti-rabbit antibody (red). Original magnification, x600. CK = cytokeratin; CTCs = circulating tumour cells; FITC = fluorescein isothiocyanate. Click here for file
