# Supplementary figures and images for: Epithelial to mesenchymal transition markers expressed in circulating tumour cells of early and metastatic breast cancer patients
Source: Breast Cancer Res. 2011 Jun 10;13(3):R59. doi: 10.1186/bcr2896 (PMC3218948; doi:10.1186/bcr2896)

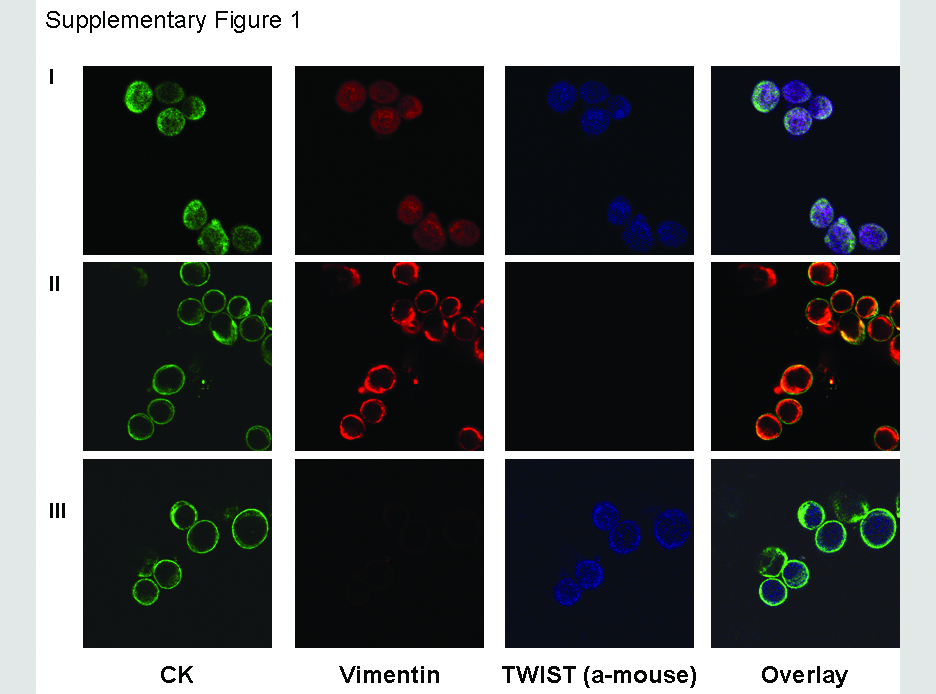

Supplement: Additional file 1 — Triple-staining control experiments in HeLa cells. Triple-staining control experiments in HeLa cells analysed using a confocal laser-scanning microscope. (I) Representative confocal laser-scanning photomicrographs of positive controls for Twist anti-mouse antibody. HeLa cells were stained with pan-CK A45-B/B3 antibody/Zenon Alexa Fluor 488 (green)/Twist anti-mouse/Alexa Fluor 633 anti-mouse IgG (blue)/vimentin anti-rabbit antibody/Alexa Fluor 555 anti-rabbit antibody (red). Original magnification, x500. (II) Negative control for Twist. HeLa cells stained with pan-CK A45-B/B3 antibody/Zenon Alexa Fluor 488 (green)/Alexa Fluor 633 anti-mouse IgG (blue)/vimentin anti-rabbit antibody/Alexa Fluor 555 anti-rabbit antibody (red). Original magnification, x500. (II) Negative control for vimentin. HeLa cells were stained with pan-CK A45-B/B3 antibody/Zenon Alexa Fluor 488 (green)/Twist anti-mouse antibody/Alexa Fluor 633 anti-mouse antibody (blue)/Alexa Fluor 555 IgG isotype antibody (red). Original magnification, x500. CK = cytokeratin; HeLa = patient with cervical adenocarcinoma, from which the cell line was derived; IgG, immunoglobulin G. [file bcr2896-S1.TIFF]

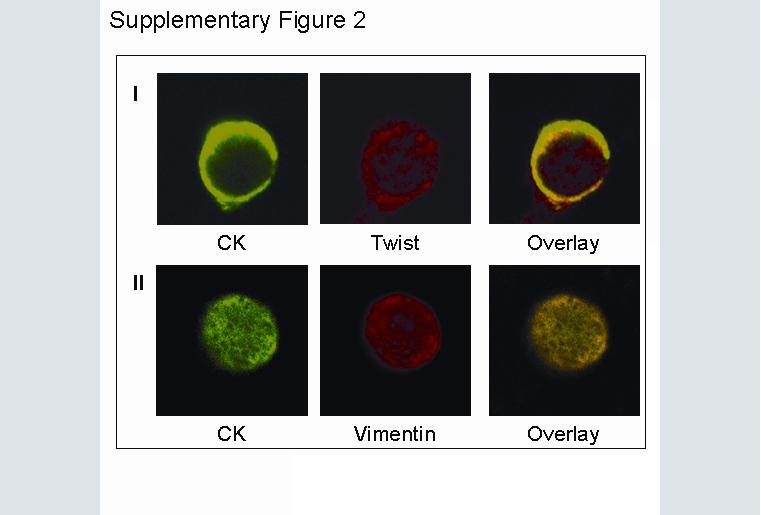

Supplement: Additional file 2 — Twist and vimentin expression in CTCs of breast cancer patients. Representative confocal laser-scanning microscopic images of Twist-or vimentin-expressing CTCs of breast cancer patients. (I) Representative confocal laser-scanning microscopic images of breast cancer patients' cytospin double-stained with monoclonal pan-CK (A45-B/B3)/Alexa Fluor 488 anti-mouse antibody (green)/polyclonal Twist anti-rabbit antibody and Alexa Fluor 555 anti-rabbit antibody (red). Original magnification, x600. (II) Representative images of cytospin double-stained with monoclonal pan-CK (A45-B/B3)/Alexa Fluor 488 anti-mouse antibody (green)/polyclonal vimentin anti-rabbit antibody and Alexa Fluor 555 anti-rabbit antibody (red). Original magnification, x600. CK = cytokeratin; CTCs = circulating tumour cells; FITC = fluorescein isothiocyanate. [file bcr2896-S2.TIFF]
